# A Single Dose of Yellow Fever Vaccine Provides Long-Term Immunity in Japanese Travelers

**DOI:** 10.3390/vaccines13070675

**Published:** 2025-06-24

**Authors:** Shinji Fukushima, Chang Kweng Lim, Atsuo Hamada

**Affiliations:** 1 Travellers’ Medical Center, Tokyo Medical University Hospital, 6-7-1 Nishi-Shinjuku, Shinjuku-ku 160-0023, Tokyo, Japan; 2 Department of Virology 1, National Institute of Infectious Diseases, 1-23-1 Toyama, Shinjuku-ku 162-8640, Tokyo, Japan

**Keywords:** yellow fever (YF) vaccine, anti-yellow fever virus (YFV)-neutralizing antibody, immunogenicity, long-term immunity

## Abstract

Yellow fever (YF) is an acute hemorrhagic zoonotic disease that causes severe liver damage, renal failure, and hemorrhagic shock. No antiviral treatment is available; thus, vaccination is a critical preventive measure. Although the World Health Organization (WHO) revised the guidelines regarding the need for booster vaccination for YF with the rationale that a single vaccination provides sufficient long-term immunogenicity, no studies have evaluated long-term immunity in Japanese adults who received a single dose of YF vaccine. This study evaluated the long-term persistence of immunogenicity in Japanese adults vaccinated with the YF vaccine. This observational study enrolled Japanese adults who received a single YF vaccination >5 years previously. Blood samples were collected after confirming eligibility for the study. The serum levels of anti-yellow fever virus (YFV)-neutralizing antibodies were measured using the 50% plaque reduction neutralization test (PRNT_50_). The 65 participants comprised 35 males and 30 females, with a median age at vaccination of 34 years. The time between YF vaccination and registration was between 5 and 26 years. All participants remained seropositive even after a long time. Statistical analysis showed no correlation between the time elapsed since YF vaccination and PRNT_50_. Our results indicate that a single dose of YF vaccine provides adequate long-term immunity in Japanese adults and that booster vaccinations are not routinely required. These findings strongly aid in the development of travel medicine guidelines and the optimization of vaccination strategies by reducing the usage of medical resources and simplifying the health requirements for travelers.

## 1. Introduction

Yellow fever (YF) is an acute hemorrhagic zoonotic disease caused by the yellow fever virus (YFV), a positive-sense single-stranded RNA virus of the genus *Orthoflavivirus*, family *Flaviviridae*, and is endemic to tropical and sub-tropical regions of South America and sub-Saharan Africa [1,2]. Transmission occurs through the bite of infected mosquitoes, primarily by the mosquito vector *Aedes aegypti*. The disease can progress dramatically, leading to severe liver damage, renal failure, and hemorrhagic shock, with a case fatality rate of 31–47% [3].

YF is a severe acute disease for which no antiviral treatment is available, making vaccination the most crucial preventive measure. The live-attenuated 17D vaccine has been used for a long time and has proven to be safe and effective [4]. Previously, booster doses of the YF vaccine have been recommended every 10 years for those at risk of exposure, including people living in endemic countries and travelers [5]. However, in 2013, based on the recommendations of the Strategic Advisory Group of Experts on Immunization (SAGE), the World Health Organization (WHO) revised its position on YF vaccination, concluding that a single dose confers lifelong protection, thereby eliminating the need for revaccination [6]. As a result, the International Health Regulations were updated in 2016 regarding the validity of vaccination certificates. Since then, the adequacy of the single dose for lifelong protection has been a topic of debate in various studies [7,8,9,10,11]. These studies showed that the period of protection after YF vaccination is long, but there is evidence that anti-YFV-neutralizing antibody titers decline over time and reach levels that can be considered seronegative in at least some cases of people who received a single dose of YF vaccine [12]. This situation is of more concern for people who have lived in endemic areas and those with lifelong exposure to YFV [13].

Japan is one of the YF-non-endemic countries, and Japanese people receive the YF vaccine as a travel-related vaccine when visiting YF-endemic countries. For residents of YF-non-endemic countries who are not normally exposed to YFV, it is important to retain antibodies against YFV during travel. In Japan, vaccination against YF is performed by administering 0.5 mL of the 17D-204 vaccine subcutaneously. Short-term data on immunogenicity following YF vaccination are available [14]. However, data regarding the long-term immunogenicity of YF vaccines are lacking. This study evaluated long-term immunogenicity in Japanese adults who received a single dose of the YF vaccine.

## 2. Materials and Methods

### 2.1. Study Design and Trial Registration

This observational study was conducted from 8 December 2020 to 30 September 2023 at Tokyo Medical University Hospital in Japan. The study was registered with the Clinical Trial Registry (UMIN000040526) on 1 August 2020 before Japanese adult participants were registered for the study.

### 2.2. Participants

Eligible participants were Japanese adults aged 20 years or older with a history of YF vaccination more than 5 years earlier. Exclusion criteria were age less than 20 years at registration, a history of multiple doses of YF vaccine, or YF vaccination within 5 years.

### 2.3. Study Procedures

All participants signed an informed consent form before participating in this study. Blood samples were collected after confirming eligibility for the study. Participants completed a questionnaire that included information on their sex, date of birth, and YF vaccination history obtained from the immunization records.

The blood was allowed to clot, and the serum was separated by centrifugation at 1500× *g* for 10 min. Serum samples were stored at ≤−20 °C. All blood samples were shipped to the National Institute of Infectious Diseases in Tokyo, Japan, and anti-YFV-neutralizing antibody titers were determined.

### 2.4. Measurement of Anti-YFV-Neutralizing Antibody Titers

The neutralizing antibody titer against YFV was determined using a 50% plaque reduction neutralizing antibody test (PRNT_50_), which has been widely used in previous studies on YF vaccine evaluation [15,16,17,18]. Serum samples were heat-inactivated at 56 °C for 30 min and diluted 2-fold from 1:10 to 1:2560. Equal amounts of the inactivated serum and attacking YFV (strain 17D; 2.0 × 10^3^ plaque forming unit (PFU)/mL) were mixed and inoculated in duplicate into 12-well plates of confluent Vero cells (strain 9013) at 50 PFU/well [19,20]. After inoculation, the Vero cells were overlaid with a semi-solid medium containing 1% methyl cellulose and incubated for 5 days. The highest dilution of serum that showed plaque reduction of 50% or more was used as the neutralizing antibody titer.

### 2.5. Statistical Analysis

No hypothesis testing was conducted in this study; descriptive statistics were used to summarize the data. Seropositivity, geometric mean titer (GMT), and 95% confidence interval (CI) were calculated. Seropositivity was defined as the proportion of participants with neutralizing antibody titers ≥ 1:10 [21].

Antibody prevalence and geometric mean titer (GMT) were compared among subgroups based on sex, age at yellow fever (YF) vaccination, and the time elapsed between YF vaccination and study entry. Seroprevalence comparisons were performed using Fisher’s exact test, while GMT comparisons were conducted using the Wilcoxon rank-sum test. Correlation analysis was carried out using Spearman’s rank correlation coefficient. A two-tailed *p* value of less than 0.05 was considered statistically significant.

All statistical analyses were conducted using EZR [22], a modified version of the R commander designed to add statistical functions frequently used in biostatistics (JICHI Medical University, Tochigi, Japan).

### 2.6. Ethical Considerations

The study was conducted in compliance with the principles of the Declaration of Helsinki, and approved by the Institutional Review Board of the Tokyo Medical University (T2020-0269) on 8 December 2020. All participants provided written informed consent before enrollment.

## 3. Results

### 3.1. Characteristics

The 65 participants comprised 35 males and 30 females, with a median age at YF vaccination of 34 years (range 19–64 years, interquartile range IQR 28–43 years), and a median age at registration of 45 years (range 24–70 years, IQR 38–55 years). The elapsed time between YF vaccination and registration ranged from 5 to 26 years (Table 1). No participants were immunocompromised. The number of participants who lived for an extended period in countries at risk for yellow fever was 23, with 14 in Africa and 9 in South America.

### 3.2. Protection and GMT After a Single Dose of YF Vaccine

All participants remained seropositive even after a long time. The neutralizing antibody titers among the participants ranged from 1:10 to 1:640. There were no significant differences in GMT based on sex or age groups at the time of yellow fever vaccination. The geometric mean antibody titers over time since yellow fever vaccination were 68.3 (95% confidence interval: 49.8–93.8) at 5–9 years post-vaccination and 43.9 (95% confidence interval: 31.0–61.9) at 10–25 years post-vaccination, with no significant differences observed (Table 1).

### 3.3. Relationship Between Antibody Titers and Time Elapsed Since YF Vaccination

**Figure 1:**
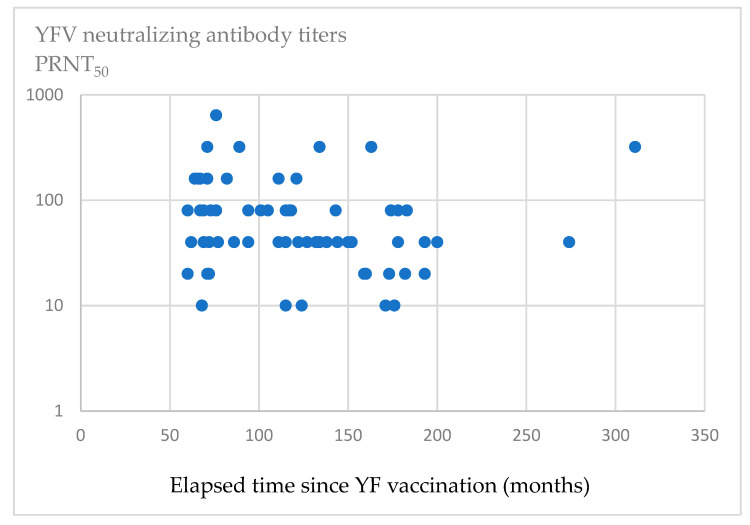
A scatter plot of anti-YFV-neutralizing antibody titers in relation to time elapsed since vaccination. The horizontal axis represents months elapsed since the last vaccination, and the vertical axis represents the anti-YFV-neutralizing antibody titers obtained by PRNT_50_.

Figure 1 shows a scatter plot of the anti-YFV-neutralizing antibody titer in relation to the time elapsed since YF vaccination. The spearman correlation coefficient was −0.246 (*p* < 0.05). No correlation was found between the months elapsed since YF vaccination and anti-YFV-neutralizing antibody titers.

## 4. Discussion

Research on the duration of immunity after YF vaccination is extremely important for travel medicine and public health policies. In particular, scientific evidence is needed to determine the necessity and timing of booster vaccinations when designing and implementing vaccination programs for travelers and residents in endemic areas.

This study evaluated the long-term duration of immunity against YFV in Japanese adults after YF vaccination. This study confirmed that anti-YFV neutralizing antibody positivity was maintained, even in adults vaccinated more than 10 years previously. Notably, there was no significant difference in geometric mean titers between those who were vaccinated within the past 10 years and those who were vaccinated more than 10 years earlier, suggesting that the long-term immunogenicity of the vaccine remains strong. A study from Korea, a YF-non-endemic country like Japan, reported that anti-YF antibody titers were decreased in individuals who had been vaccinated a long time previously [23]. Our data showed no correlation between the number of years elapsed since YF vaccination and anti-YFV-neutralizing antibody titers. In our study, 23 individuals had previously stayed for an extended period in countries where YF was endemic, suggesting that they might have been naturally exposed to the YF virus. However, the neutralizing antibody titers in all results were ≤1:640, suggesting that the participants in this study were unlikely to have had recent natural YFV infections. The remaining 43 individuals had not stayed long-term in YF endemic countries, so the likelihood of natural infection was also considered low.

The YF vaccine is thought to induce long-term immunity because it induces T cells and maintains antibody titers from B cells for a long period [24,25]. For immunocompetent participants in our study, the routine booster administration of YF vaccine is not considered necessary. Breakthrough infections of YF following YF vaccination are reported to be rare [26].

The findings of this study are extremely important for travel medicine guidelines, particularly for adults from non-endemic countries traveling to areas endemic for YF. The data support the idea that a single dose of YF vaccine provides long-term immunity, which is essential when optimizing vaccination strategies. In addition, the findings save medical resources and simplify health requirements for travelers. However, some research results suggest that a booster administration of YF vaccine should be considered for certain groups [27,28,29,30,31].

This study had several limitations. First, the sample size of individuals vaccinated more than 10 years ago was small, so caution is needed when generalizing the results. It is also important to note the small number of elderly and immunocompromised participants, because their immune responses may vary significantly [32,33,34]. In addition, the study did not include children, so long-term immunity in the pediatric population could not be evaluated [35]. A booster dose should be considered for certain travelers planning to travel to high-risk areas based on their immune competence and time since vaccination [32]. In particular, booster doses may be required for people with HIV and children under 2 years old [36].

## 5. Conclusions

In conclusion, this study showed that Japanese adult travelers can maintain serological protection against YF for a long period after vaccination. This indicates that adults outside areas endemic for YF can acquire immunity for more than 10 years with a single vaccination regimen. The study supports the 2013 WHO SAGE guidelines, which state that immunocompetent individuals do not require routine booster doses for lifelong protection. This could have significant implications for public health policies and the management of YF vaccination programs worldwide [37,38].

## Figures and Tables

**Table 1:** Seropositive rate and GMT.

Characteristics	N (%)	Seroprevalence % (95%CI)	GMT (95%CI)
Total	65	100 (94.5–100)	55.7 (44.1–70.3)
Sex			
Male	35 (53.85)	100 (90.0–100)	60.6 (44.2–83.4)
Female	30 (46.15)	100 (88.4–100)	50.4 (35.1–72.4)
		*p* = 1	*p* = 0.435
Age at YF vaccination (years)			
<40	40 (61.5)	100 (91.2–100)	51.9 (39.1–68.9)
≥40	25 (38.5)	100 (86.3–100)	62.3 (40.6–95.7)
		*p* = 1	*p* = 0.449
Age at registration (years)			
<40	35 (53.85)	100 (81.5–100)	58.8 (38.9–88.9)
≥40	30 (46.15)	100 (92.5–100)	54.5 (40.7–72.9)
		*p* = 1	*p* = 0.775
Time elapsed since YF vaccination (years)			
5–9	35 (53.85)	100 (90.0–100)	68.3 (49.8–93.8)
10–26	30 (46.15)	100 (88.4–100)	43.9 (31.0–61.9)
		*p* = 1	*p* = 0.059

YF, yellow fever; GMT, geometric mean titer.

## Data Availability

The original contributions presented in this study are included in the article. Further inquiries can be directed to the corresponding author(s).

## Abbreviations

The following abbreviations are used in this manuscript:

YF	Yellow fever
YFV	Yellow fever virus
WHO	World Health Organization
PRNT50	50% plaque reduction neutralizing antibody test
IQR	Interquartile range
GMT	Geometric mean titer
CI	Confidence interval
PFU	Plaque-forming unit
